# Molecular tools to support metabolic and immune function research in the Guinea Fowl (*Numida meleagris*)

**DOI:** 10.1186/s12864-015-1520-6

**Published:** 2015-05-07

**Authors:** Carl E Darris, James E Tyus, Gary Kelley, Alexander J Ropelewski, Hugh B Nicholas, Xiaofei Wang, Samuel Nahashon

**Affiliations:** College of Agriculture, Human and Natural Sciences, Tennessee State University, Nashville, Tennessee USA; Pittsburgh Supercomputing Center, Carnegie Mellon University, Pittsburgh, Pennsylvania USA

**Keywords:** Guinea fowl, RNA-seq, De novo assembly, Metabolic function, Immune function

## Abstract

**Background:**

Guinea fowl (*Numidia meleagris*) production as an alternative source of meat and poultry has shown potential for economic viability. However, there has been little progress in characterizing the transcriptome of the guinea fowl. In this study RNA-sequencing and de novo transcriptome assembly of several Guinea fowl tissues (pancreas, hypothalamus, liver, bone marrow and bursa) which play key roles in regulating feed intake, satiety, and immune function was performed using Illumina’s Hi-Seq 2000.

**Results:**

74 million sequences were generated and assembled into 96,492 contigs using the Trinity software suite. Over 39,000 of these transcripts were found to have *in silico* translated protein sequences that are homologous to chicken protein sequences. Gene ontology analysis uncovered 416 transcripts with metabolic functions and 703 with immune function.

**Conclusion:**

The transcriptome information presented here will support the development of molecular approaches to improve production efficiency of the guinea fowl and other avian species.

**Electronic supplementary material:**

The online version of this article (doi:10.1186/s12864-015-1520-6) contains supplementary material, which is available to authorized users.

## Background

Population projections and high production costs necessitate research to identify and develop alternative sources of meat and poultry. The guinea fowl (GF) is a provocative poultry alternative due to its superior nutritional value and economic potential [1]. Over the past decade poultry research has benefited greatly from advances in sequencing technology, with the genome and various transcriptome projects of both the chicken and the turkey being completed. While there are efforts to generate similar data in non-model avian species [2-4], to date there is very limited genetic information available to aid the effort of bringing GF meat and poultry products into mainstream consumption at a reasonable price [5-17]. The application of transcriptome data generated through RNA-sequencing has the potential to provide clues that will increase our understanding of the metabolic regulation of appetite, feed utilization, immune function, growth and overall production performance of guinea fowl.

Transcriptome analysis of the guinea fowl will provide fundamental data needed to develop species-specific management tools, such as feed and disease prevention regimens. The coupled selection of metabolic and immune function traits is of the utmost importance, as continual selection for single metabolic or growth traits have had a detrimental effect on immune function in poultry [18]. At first glance this may seem a bit surprising because a rapid growth rate is normally associated with good overall health, to include immune function. It has been shown however, that trait selection imposes an energy trade-off within organisms [18]. Previous studies have investigated the trade-offs between immune function versus reproduction, production traits, and growth in birds, sheep, and insects respectively [19-21]. Collectively these studies have shown that immune function as a trait is dynamic, energetically costly and requires optimization in concert with other selected traits. Interestingly, van der Most et al. found that while the selection for growth was detrimental to immune function, the selection of immune function does not comprise growth performance in poultry [18]. These findings open the door for the establishment of high-performance lines of poultry by allowing for the selection of disease resistance and growth simultaneously.

Attaining these goals requires a wealth of genetic information and an in-depth understanding of the role played by each gene involved in the regulation of metabolism, satiety, feed utilization, conversion and metabolism, and immune function. De-novo sequencing and transcriptome assembly of the guinea fowl pancreas, hypothalamus, liver, bone marrow and bursa was performed as a first step to developing this required pool of data unique to the GF. Such information is essential in revealing new metabolic pathways that may be utilized to improve growth and production performance of both traditional and non-traditional poultry such as chickens and guinea fowl, respectively.

The aim of this study was to **(i)** perform targeted de novo assembly of the guinea fowl transcriptome of the pancreas, liver, hypothalamus, spleen, bursa and bone marrow; **(ii)** to compile a database of functional annotations for the assembled guinea fowl transcriptome; **(iii)** to perform comparative analysis of the assembled guinea fowl transcriptome using chicken and turkey protein databases; **(iv)** to identify guinea fowl transcripts with metabolic and immune function.

## Results and discussion

In this study we set out to develop a transcriptome library that would reveal unique gene sequences to aid the understanding of key and unique metabolic and immune processes in the guinea fowl. Application of the data generated in this study will serve to improve the production performance of guinea fowl and other related avian species.

### Transcriptome assembly

As mentioned above, the original 74 million Illumina reads (4.9GB of raw data) was reduced to approximately 53 million reads through trimming and filtering. These remaining reads were assembled into 96,491 contigs (Additional file 1). The GC content per contig was 52% for both the pancreas and liver samples and 49% for the hypothalamus and bursa/bone marrow samples.

**Figure 1:**
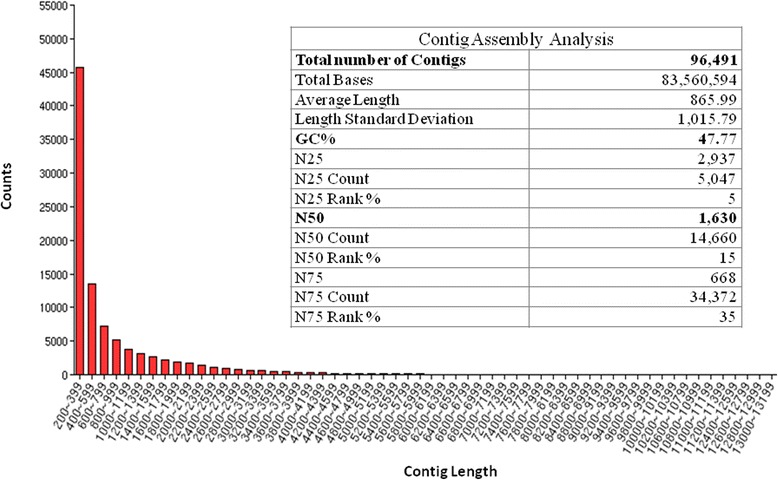
Analysis of Contig Assembly. Contig assembly resulted in 96,491 contigs with a minimum length of 200 bases, a mean length of 865 bases and a N50 value of 14,660.

The average length of the assembled contigs was 866 bases with a N50 of 1630 nucleotides. Over 45,000 of these assembled contigs ranged from 200–399 base pairs (Figure 1). The large number of short contigs appears to partially be the result of single end sequencing and partially due to the assembly method. While Trinity tends to recover more correct transcripts overall than other methods, it also tends to recover a great deal of partial transcript sequences [22].

### Functional annotation

A total of 47,079 contigs assembled by Trinity returned statistically significant (1.0e^-5^) hits after blastp and BlastX searches against the SwissProt section of UniProt and the entire Chicken and Turkey proteomes.

**Figure 2:**
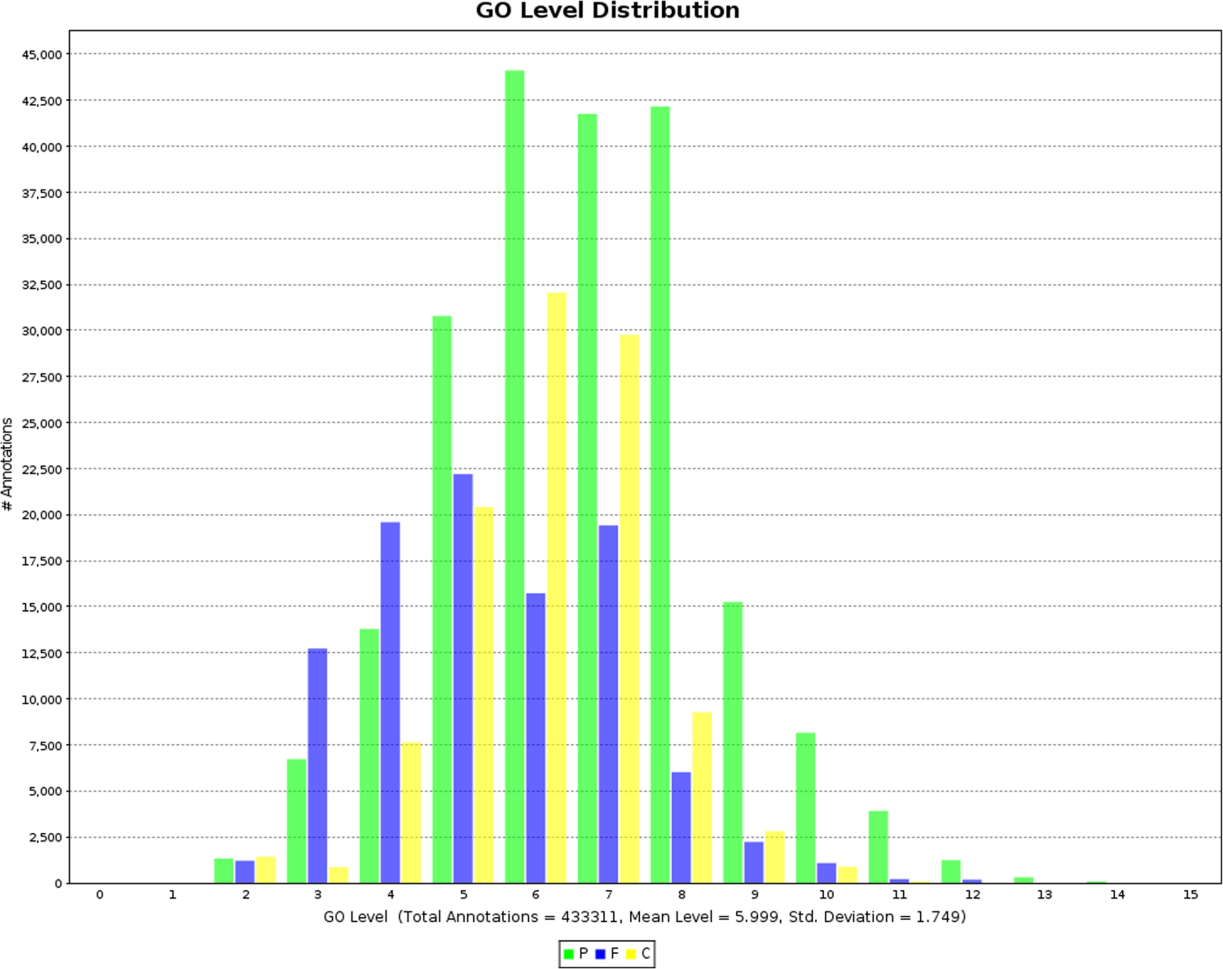
Distribution of GO Annotation. In total 433331 annotations across all categories (P: biological processes, C: cellular components, F: molecular function) were assigned to the GF contigs with the mean GO level of 6.

**Figure 3:**
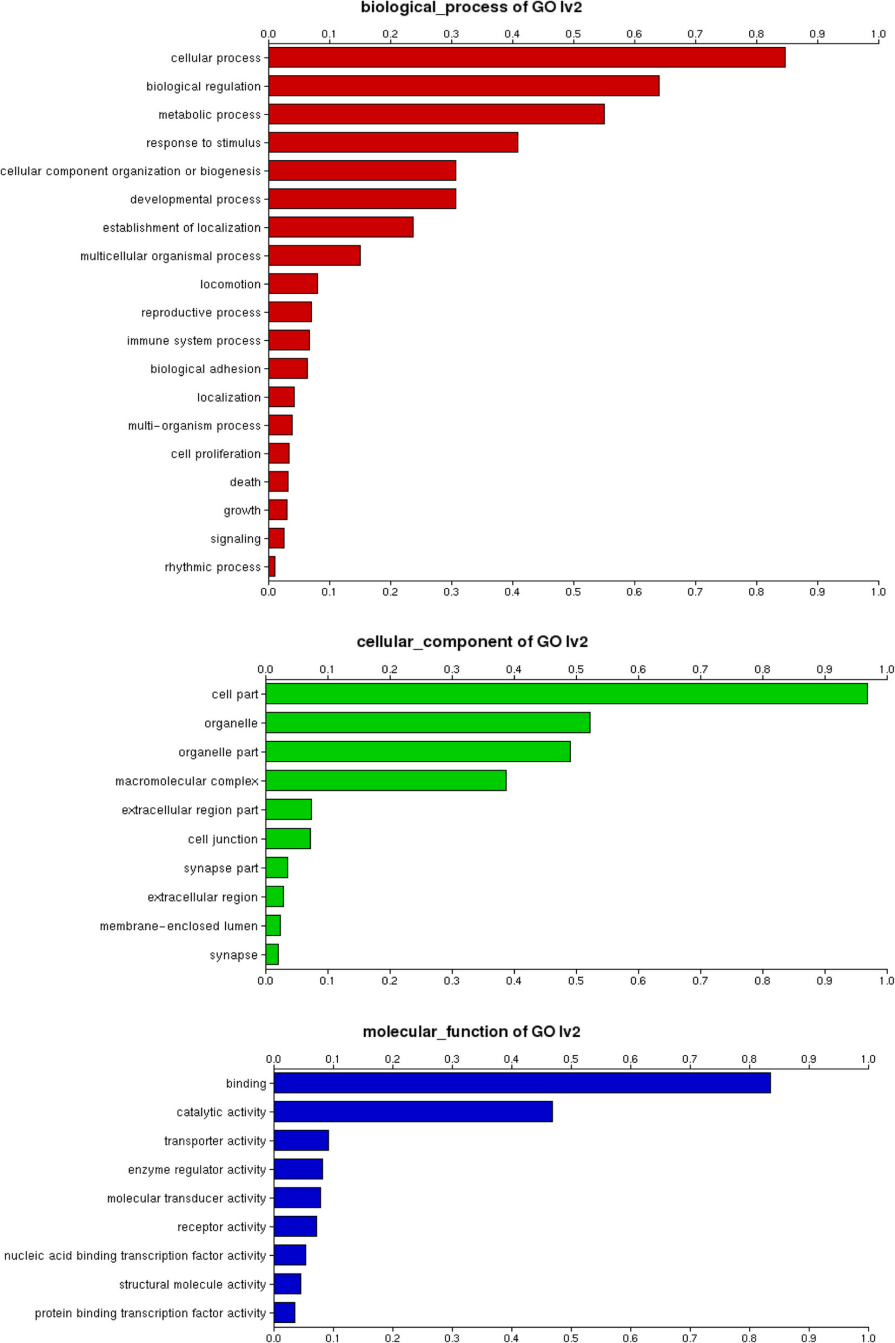
Distribution of Level 2 GO Terms. These bar charts illustrate the distribution of GO terms categorized as biological process, cellular components or molecular function assigned to GF contigs. Distribution within the molecular function category indicates some role in binding for over 80% of the contigs.

**Figure 4:**
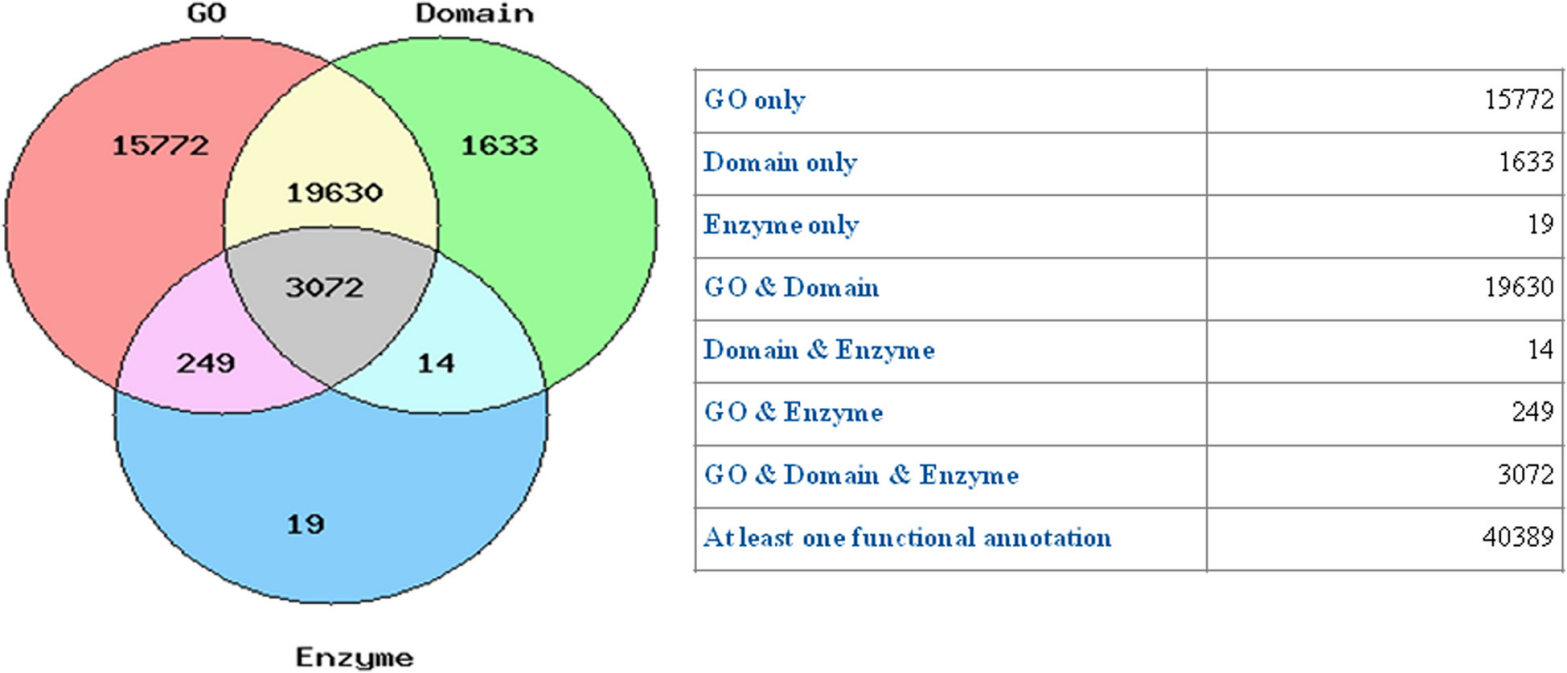
GF contigs Annotation results from Fast Annotator. Depicted in this venn diagram are the annotation results from Fast Annotator software which assigned a total of 38,723 GO terms, detected 24,349 domains, and identified 3354 homologous enzyme-related sequences. 3,072 contigs had all three levels of annotation.

The Gene Ontology terms assigned to the contigs were well distributed between the categories of biological process, cellular component and molecular function, with a mean level of 6 (Figures 2 and 3). Of these, 38,673 were assigned at least one Gene Ontology term. In addition 3,354 were assigned an enzyme annotation (Figure 4). Transcripts that had e-values rising above 1e^-10^ were annotated. This Transcriptome Shotgun Assembly project has been deposited at DDBJ/EMBL/GenBank under the accession GBYG00000000. The version described in this paper is the first version, GBYG01000000.

Gene coverage analysis for the annotated sequences revealed that 5,796 or 23.5% of the sequences that yielded hits covered more than 50% of the gene to which they shared homology. A total of 866 sequences showed 100% gene coverage. The predicted open reading frames encoded by the sequences analyzed ranged from 200 to 4000 bases in length of with the majority falling between 100 to 200 bases. Due to the low coverage of the reads and lack of replicates a differential expression analysis between the individual tissues was not performed. FastAnnotator identified 24,349 domains in the query nucleotide sequences with coverage greater than 50% by searching against domain models from the Conserved Domains Database at an expectation value (e-value) limit of 0.01 (Figure 4).

### Identification of orthologus contigs

Forty percent of the contigs were homologous with protein sequences deposited in the blastp database for chicken (39,376) and turkey (39,474) (Additional file 2). GO terms were used to mine the Trinotate output (Additional file 3) and identify contigs annotated with metabolism or immune function. In total 416 transcripts with metabolic function and 703 with immune function were uncovered (Tables 1 and 2).

**Table 1:** **Metabolic function related GO term search of Trinotate output**

**Metabolic Function Terms**		**Contigs**
GO:0005976	polysaccharide metabolic process	4
GO:0006109	regulation of carbohydrate metabolic process	2
GO:0006629	lipid metabolic process	252
GO:0008152	metabolic process	151
GO:0016052	carbohydrate catabolic process	6
GO:0019538	protein metabolic process	4
GO:0044262	cellular carbohydrate metabolic process	13
GO:0051246	regulation of protein metabolic process	13

**Table 2:** **Immune function related GO term search of Trinotate output**

**Immune Function Terms**		**Contigs**
GO:0045087	innate immune response	445
GO:0006959	humoral immune response	37
GO:0006955	immune response	244
GO:0050776	regulation of immune response	36
GO:0002682	regulation of immune system	7
GO:0050778	positive regulation of immune response	8
GO:0033025	regulation of mast cell apoptotic	4
GO:0045637	regulation of myelo cell differentiation	3
GO:0002683	negative regulation of immune system	1
GO:0034121	regulation of protein metabolic process	2

## Conclusion

Most of the desired traits in farm animals such as body mass, production yield, and disease resistance are quantitative. Over the centuries traditional selective breeding of superior individuals has resulted in the marked enhancement of production traits based on phenotypic expression of desirable traits [23]. These traditional means of breed improvement through phenotypic selection have led to enhancement of economically important traits in cattle, sheep, pigs, poultry and other livestock [24]. One drawback however, is that traditional methods of breeding are limited in their ability to select for traits that are difficult to measure such as fertility, longevity and disease resistance. These traits do not lend themselves to such dramatic improvement through selection alone [23]. It is in these areas that transcriptomic data has the most potential for direct and immediate application.

By focusing on those genes that code for proteins related to traits of interest, poultry scientists have gleaned and applied genetic information to increase the production and performance of chicken and other poultry birds. The transcriptomic data set presented in this study contributes to the genomic and proteomic resources available for GF development. These genetic tools will support the progression of molecular approaches to improve the profitability of guinea fowl production. Our results show that sixty percent of the contigs were non-homologous with protein sequences deposited in the blastp database for chicken. The continued curation of this putative set of novel GF genes is essential for downstream comparative analysis, expression profiling, functional studies and trait selection across avian species.

## Methods

### Animals and RNA preparation

The pancreas, liver, hypothalamus, bone marrow and bursa were harvested from an eight week old male guinea fowl which was housed under a 12-hour light/dark cycle and fed a diet comprising of 3,340 kcal of metabolizable energy/kg of diet and 23% crude protein. Feed and water were provided for *ad libitum* consumption. Animal use for this study was approved by Tennessee State University Institutional Animal Care and Use Committee (IACUC). Following sacrifice by cervical dislocation, liver (approximately 5 g from the mid-portion of the anterior sub-segment of the right lobe), pancreas (approximately 2 grams of tissue from the duodenal loop), tibial bone marrow, and bursa (whole organ) were removed and submerged in an RNA stabilization solution (pH 5.2) containing 0.5 M EDTA, 1 M sodium citrate, and 700 g ammonium sulfate dissolved in ultrapure water overnight at 4°C. Whole heads were flash-frozen in liquid nitrogen. Subsequently, hypothalami were excised by micro-dissection and submerged in an RNA stabilization solution. All tissue samples were stored at −80°C until use. Total RNA was isolated from each tissue using Qiagen’s RNeasy® Mini Kit according to the manufacturer’s protocol. (Qiagen, Valencia, CA) Total RNA concentrations were determined via NanodropTM Spectrophotometer (Thermo Scientific; Wilmington, DE). Each sample was diluted to 50 ng/μl, separated into 50 μl aliquots and immediately frozen at −80°C. Sample quality was evaluated by visual inspection of a 1% formaldehyde gel ran at 100 volts for 1 hour. Gel images were captured using the Kodak Gel Logic 1500 Imaging System (Kodak; Rochester, NY). Experion™ Automated Electrophoresis System (Bio-Rad; Hercules, CA) was used to confirm RNA quality according to the manufacture’s guidelines. Sample quality was also confirmed using a BioAnalyzer (Agilent; Santa Clara, CA). The resulting RIN values for the hypothalamus, pancreas, liver and bursa/bone marrow samples were 9.5, 7.9, 5.3 and 5.5 respectively.

### Library construction and iillumina sequencing

The cDNA library construction was conducted at the Vanderbilt University’s Genomic Sciences Resource Center (VUGSR), Nashville, TN (VUGSR). During library construction, mRNA was isolated from 100 ng of total RNA followed by fragmentation, 1^st^ then 2^nd^ strand cDNA synthesis. The cDNA was end-repaired, size selected and then ligated to adapter sequences. The cDNA libraries were multiplexed and sequenced in one lane using Illumina’s Hi-Seq 2000 (Illumina, Inc., San Diego, CA) single end read sequencing platform. The sequencing run produced approximately 74 million single end reads with average length of 101 bp. The resulting reads were de-multiplex and reported as separate runs and deposited in the National Institutes of Health (NIH) Short Read Archive (http://ncbi.nlm.nih.gov/sra) (Pancreas: SRS584523, Hypothalamus: SRS413447, Liver: SRS585609, Bone Marrow/Bursa: SRS586251).

### Assembly, annotation, and gene ontology analysis

Prior to assembly, all reads were run through quality control procedures to ensure that Illumina adapters were removed and that only high quality data was used in the assembly. The FastQC program was used to perform an examination of the reads. Based on those results, tools in the fastx toolkit were used to remove Illumina adapters, performing end trimming of reads, as well as filtering reads out of the dataset that had average quality values < 30 (sup. Figure 1). After these trimming and filtering procedures, approximately 54 million reads remained for assembly.

Assembly and annotation was performed on Blacklight, a SGI UV 1000 cc-NUMA shared-memory system available to U.S. academic researchers through the NSF XSEDE program (www.xsede.org).

Transcripts were assembled de novo using Trinity (r2012-08-14) (available at http://trinityrnaseq.sourceforge.net/) using the default settings [22,25]. To ensure a uniform transcriptome reference across the datasets, all reads were pooled for assembly then the datasets were individually aligned back to the reference transcriptome.

The transcriptome produced was annotated using Trinotate (r2013-08-26) (available at http://trinotate.sourceforge.net). The Trinotate suite provides for the functional annotation of de novo assembled transcriptomes and makes use of several annotation techniques including blastp/blastx database searches against reference sequence databases, PFAM domain searches, and various signal predictions. Trinotate integrates this initial annotation information into a relational database that includes reference information from Uniprot, and eggNOG/GO Pathways databases. Due to the modular design of the system and the use of an SQL database, the system was modified to include information contained within the complete proteome sequences of both the chicken and turkey.

The assembled transcripts were also submitted to FastAnnotator for comparative annotation and identification of domains and potential enzyme functions. Fast Annotator (available at fastannotator.cgu.edu.tw) was used to analyze the distribution of gene ontology terms, develop graphical representations of the data set and for enzyme identification [26]. Gene Ontonlogy (GO) terms used to identify associated with metabolic functions: GO:0005976, GO:0006109, GO:0006629, GO:0008152, GO:0016052, GO:0019538, GO:0044262,GO:0051246. GO terms used to identify associated with immune functions: GO:0045087, GO:0006959, GO:0050776, GO:0002682, GO:0050778, GO:0033025, GO:0045637, GO:0002683, GO:0034121.

### Availability of supporting data

The data sets supporting the results of this article are included within the article and its supplemental files.

## Additional files

Additional file 1:
**GF_Trinity Output Fasta.**
Additional file 2:
**Blastp XLS.**
Additional file 3:
**Trinotate Annotation Report.**

## Abbreviations

cDNA: Complimentary Deoxy-Ribose Nucleic Acid
GC: Guanine Cytosine
GF: Guinea Fowl
GO: Gene Ontology
mRNA: messenger Ribose nucleic acid
PFAM: The Pfam database is a large collection of protein families
RNA: Ribose Nucleic Acid
RNA-seq: Ribose Nucleic Acid Sequencing
SQL: Structured Query Language
